# The Wrong Use of Words

**Published:** 1895-05

**Authors:** 


### Editorial.

### THE WRONG USE OF WORDS.

THE younger writers of the present period and perhaps in all epochs of the world's history have been prone to luxuriate in prolixity of words and superfluity of expression. The art of simplicity in writing seems difficult of attainment and perhaps few of us have a comprehension how very nearly this lies to clearness of thought. It is a truism to say that in order to think we must clothe the thought in words, but it is equally true that it is possible to think without words, although this may seem impossible to the ordinary observer. Words are the representatives of pictures and these were translated originally into root-forms, and through combinations with each advancing wave of civilization language was established. Science was added to science and the vocabulary of the learned became more and more specialized until those only can hope to master it who have engaged in the particular line of work it represents. Technicalities have been added to technicalities until we are burdened with a multiplicity of tongues among all civilized people known only to the few and a jargon to the many.

That this is true must be evident, and we are constantly threatened with increased additions in the multiplication of new words by the introduction of the so-called improved nomenclatures. Fortunately for the world of thought, words are not made to order, or at least are not assimilated into the language of a people or in a science in that manner.

The most important consideration in the use of language in the writer's opinion is in a direct translation of the idea into the simplest form of words attainable. It is a recognized fact that the best writers of the English tongue to-day, or at any former period, have been those using but few words of many syllables, and the nearer they have been able to express their ideas in simple forms have they been appreciated and regarded as models of style.

We have been impressed very often in professional reading with the fact that there was a positive need of a better training in this direction. We all have a tendency to a multiplication of ponderous words, as though the number of high-sounding syllables increased the force of the idea to be presented. Writings of a scientific character need above all others simple language. The difficulties that surround subjects of a recondite character make this imperative, and yet how often is this plain rule violated by writers who load their subject with technicalities, new and old, to an extent that deprives the essay of much of its value. Words are useless if they cloud ideas, and nowhere is clearness of expression more needed than in professional writing.

Akin to this and of more importance is the use of simple words in teaching. While it is true that teachers are born and not made by pedagogic training, there yet remains the possibility of producing a satisfactory teacher out of the ordinary professional man, provided the latter is absorbed with the subject and will avoid the error of over-phrasing, not inaptly expressed by having "digested a dictionary."

The use of technical terms is necessary within certain well-defined limits, but these may be easily overstepped and the writing or the teaching become a burden to the reader or the auditor. An excellent rule to follow in addressing a class is never to make use of a technical word if another can be found to explain the idea. This given and firmly engrafted on the mind it may be followed by the proper word. A valued friend and teacher always stops to explain the meaning of a technical term. This to our comprehension is not the best course to pursue. Definitions are rarely of value, as all know who have undertaken the study of a language by the aid of a dictionary. The thought must be absorbed before the word can be comprehended. Hence the folly of showering a class of young men with terms beyond their powers to utilize and expect to have them understand the subject-matter of the lecture. This is too often the mistake of professional teachers. The rule should never be forgotten to make the subject but a degree above the most ordinary mind present. Complex problems in our specialty, as in all other branches of medicine, are sufficiently difficult without increasing this by a redundancy of technical forms.

The evil is one rapidly growing in our literature, and it seems a proper time to call attention to it, especially to those fresh from college life who are apt to imagine that learning is best expressed by the use of the longest words, and that unless their ideas are thus clothed the essay will fail of proper recognition. This is a common error not by any means confined to the class mentioned.

The tendency to pedantry in writing is another defect. Use of foreign words not domesticated in our tongue, or an occasional Latin or Greek phrase, better expressed in English and which rarely represent in their use solid learning, add nothing to the value of an article, nor is the reader better able to understand, by their presence, the ideas the writer hopes to convey to the mind.

Allied to this is the search of some for a style. This has been the bane of many young and ambitious writers of all ages. It is never to be found by searching, for it is ever elusive. Style is the natural expression of the individual improved by culture. It would be as sensible to expect to acquire a good voice by searching among the singers of the world as to hope to acquire a correct style by reading the masters of word-expression in any age. As the voice can be improved by cultivation intelligently applied, in like manner the ability to embody thought can be enlarged and improved by cultivated practice.

Technical terms are important and of vital necessity in their proper place, but it is idle to expect that the young mind can absorb them at once, nor would it be desirable that they should. The professional man must learn to think in the terms he uses and until this be accomplished the idea represented cannot be absorbed, and it is just this condition that all first-year students are in to-day, a fact to be remembered by all teachers of dentistry. It is with the object of drawing attention to this subject that these ideas have been brought together, a plea for simplicity of language in all our professional relations.

